# Inhibition of Epstein-Barr Virus Lytic Reactivation by the Atypical Antipsychotic Drug Clozapine

**DOI:** 10.3390/v11050450

**Published:** 2019-05-17

**Authors:** Abbie G. Anderson, Cullen B. Gaffy, Joshua R. Weseli, Kelly L. Gorres

**Affiliations:** Department of Chemistry & Biochemistry, University of Wisconsin-La Crosse, 1725 State St., La Crosse, WI 54601, USA; anderson.abbie@uwlax.edu (A.G.A.); gaffy.cullen@uwlax.edu (C.B.G.); weseli.joshua@uwlax.edu (J.R.W.)

**Keywords:** Epstein–Barr virus, herpes viruses, lytic gene expression, Burkitt lymphoma cells, clozapine, antipsychotic drug, antiviral drug

## Abstract

Epstein–Barr virus (EBV), a member of the *Herpesviridae* family, maintains a lifelong latent infection in human B cells. Switching from the latent to the lytic phase of its lifecycle allows the virus to replicate and spread. The viral lytic cycle is induced in infected cultured cells by drugs such as sodium butyrate and azacytidine. Lytic reactivation can be inhibited by natural products and pharmaceuticals. The anticonvulsant drugs valproic acid and valpromide inhibit EBV in Burkitt lymphoma cells. Therefore, other drugs that treat neurological and psychological disorders were investigated for effects on EBV lytic reactivation. Clozapine, an atypical antipsychotic drug used to treat schizophrenia and bipolar disorder, was found to inhibit the reactivation of the EBV lytic cycle. Levels of the viral lytic genes BZLF1, BRLF1, and BMLF1 were decreased by treatment with clozapine in induced Burkitt lymphoma cells. The effects on viral gene expression were dependent on the dose of clozapine, yet cells were viable at an inhibitory concentration of clozapine. One metabolite of clozapine—desmethylclozapine—also inhibited EBV lytic reactivation, while another metabolite—clozapine-N-oxide—had no effect. These drugs may be used to study cellular pathways that control the viral lytic switch in order to develop treatments for diseases caused by EBV.

## 1. Introduction

Epstein–Barr virus (EBV) is a member of the *Herpesviridae* family and causes infectious mononucleosis. EBV was the first virus discovered to cause cancer in humans. EBV is associated with Burkitt lymphoma, Hodgkin lymphoma, gastric carcinoma, nasopharyngeal carcinoma, and post-transplant lymphoproliferative disorder. After infection with EBV, the virus maintains a lifelong latent infection within the host. The expression of a few viral genes during the latent phase allows the virus to persist. The viral life cycle alternates between two phases: the latent and the lytic phases. During the lytic phase the virus replicates and spreads among cells and hosts.

**Figure 1:**
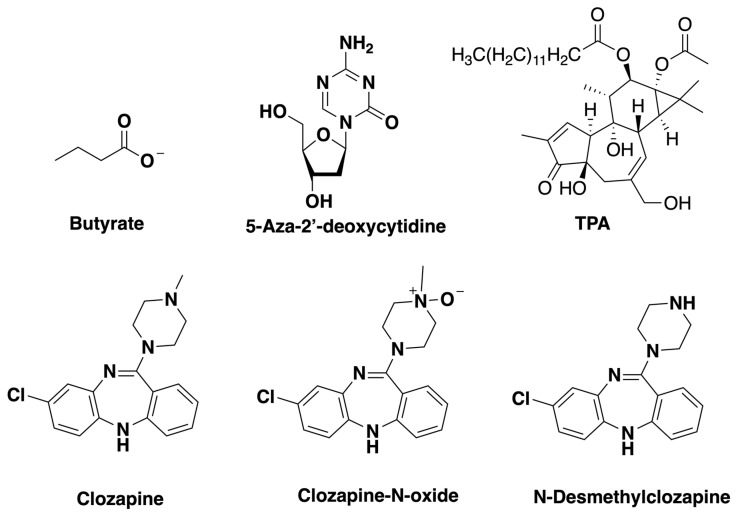
Structures of drugs tested for effects on Epstein-Barr virus (EBV) lytic reactivation: sodium butyrate (NaB), 5-aza-2’-deoxycytidine (dAzaC), 12-*O*-tetradecanoylphorbol-13-acetate (TPA), clozapine, clozapine-N-oxide, and N-desmethylclozapine.

The lytic phase of the virus can be triggered in latently infected cultured cells by various inducing agents [1]. Sodium butyrate (NaB), a short-chain fatty acid that inhibits histone deacetylases, promotes the reactivation of the lytic cycle (Figure 1) [2]. Although quite different in chemical structure from butyrate, the DNA methyltransferase inhibitors 5-azacytidine and 5-aza-2’-deoxycytidine (dAzaC), and the protein kinase C agonist 12-*O*-tetradecanoylphorbol-13-acetate (TPA) also induce the EBV lytic cycle [3]. Molecules with diverse structures inhibit reactivation of the EBV lytic cycle by these inducing agents. Some inhibitors are structurally similar to butyrate. Valproate (valproic acid, VPA) and valpromide (VPM) prevent the virus from reactivating into the lytic cycle in Burkitt lymphoma cells [4,5]. VPA and VPM are used clinically as anticonvulsant and mood-stabilizing drugs.

To determine if there is any commonality between the effects of VPA in neurological and psychological conditions and in blocking EBV reactivation, we investigated other drugs used to treat neurological conditions for effects on the EBV lytic cycle. Clozapine, a member of the dibenzodiazepine class, is used in the treatment of schizophrenia and bipolar disorder (Figure 1). Clozapine (Clozaril^TM^) was the first atypical, or second-generation, antipsychotic drug developed [6]. It is therapeutically effective at treating schizophrenic patients who are resistant to typical antipsychotic drugs [7]. We demonstrate here that clozapine and one of its metabolites inhibit the induction of EBV lytic cycle gene expression.

## 2. Materials and Methods

### 2.1. Chemicals

Sodium butyrate (NaB; >98%, Aldrich) and 5-aza-2’-deoxycytidine (dAzaC; Chem-Impex, Wood Dale, IL, USA) were dissolved in water. Clozapine (>99%, ApexBio, Houston, TX, USA), clozapine-N-oxide (>98%, ApexBio), N-desmethylclozapine (98%, Santa Cruz Biotech, Santa Cruz, CA, USA), and TPA (99%, AdipoGen, San Diego, CA, USA) were dissolved in DMSO. Drugs were used at concentrations noted in the figures and legends.

### 2.2. Cell Culture and Chemical Treatments

The HH514-16 human Burkitt lymphoma [8] and Raji cells were cultured in RPMI 1640 + glutamine supplemented with 8% FBS, penicillin (50 U/mL), streptomycin (50 U/mL), and amphotericin B (1 μg/mL). Cells were grown at 37 °C under 5% CO₂. Cells were subcultured to 3–4 × 10^5^ cells/mL two days prior to the experiment. The experiments started with 1 × 10^6^ cells/mL in RPMI 1640 supplemented with 1% FBS. Cells were harvested 24 h post-treatment. Cell death was measured by trypan blue staining and counting using a hemacytometer. In all experiments that investigated EBV reactivation, >90% of the cells were viable.

### 2.3. Lytic Reactivation by RT-qPCR

Quantitative reverse transcription polymerase chain reaction (RT-qPCR) was used to measure lytic gene expression. RNA was extracted from cells using the ReliaPrep system (Promega, Madison, WI, USA). Primers used to detect the expression of BZLF1 were AGCAGACATTGGTGTTCCAC (forward) and CATTCCTCCAGCGATTCTG (reverse); for BRLF1 they were CCATACAGGACACAACACCTCA (forward) and ACTCCCGGCTGTAAATTCCT (reverse); and for BMLF1, GGAGGAGGATGAAGATCCAA (forward) and TTTCTGGGAATCACAAACGA (reverse). The RT-qPCR utilized the iScript SYBR green RT-qPCR kit (Bio-Rad). Expression levels were normalized to 18S RNA, present at consistent levels among cells.

### 2.4. Statistical Analysis

Data are reported as the average of the number of biological replicates noted in the figure legends. The values are displayed as the mean ± standard deviation. Values are either the fold increase compared to the untreated control or the percent of maximum lytic reactivation by the inducing agent. To determine the differences among treatments, *p*-values were calculated using a paired *t*-test in R 3.3.3 of the quantitation cycle (ΔCq) values from RT-qPCR. Significant differences were considered when the *p*-value < 0.05.

## 3. Results

### 3.1. Clozapine Blocked the Induction of EBV Lytic Genes

**Figure 2:**
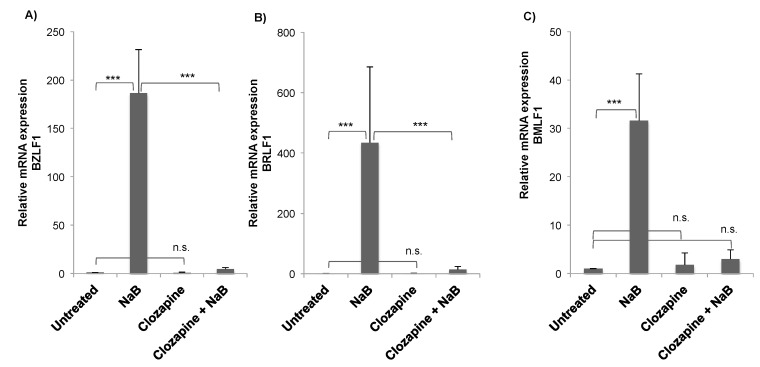
Clozapine inhibited the induction of Epstein–Barr virus (EBV) lytic gene expression. Expression of each gene, (**A**) BZLF1, (**B**) BRLF1, (**C**) BMLF1, was measured by RT-qPCR in untreated HH514-16 cells compared to treatment for 24 h with NaB (3 mM), clozapine (50 μM), or the combination of NaB and clozapine. Values are the average fold induction compared to the untreated control of four or more biological replicates. Error bars show the standard deviation. Differences with a *p*-value < 0.001 are denoted with ***, *p*-value < 0.01 with **, and not significantly different (*p*-value > 0.05) with n.s.

We investigated the response of EBV to the atypical antipsychotic drug clozapine. The experiments were performed in the HH514-16 EBV-positive Burkitt lymphoma cell line, derived from Jijoye and P3HR1 cells [8]. The degree of viral reactivation was measured by expression of the viral BZLF1 gene, an immediate early gene that encodes a transcription transactivator [9]. The expression of BZLF1 initiates the reactivation of the Epstein–Barr virus lytic cycle. Treatment of cells with the known inducing agent NaB for 24 h caused an approximately 200-fold increase in BZLF1 expression compared to untreated cells (Figure 2A). Clozapine alone did not induce BZLF1 expression or decrease basal levels of expression. When clozapine (50 μM) was added to cells with NaB, BZLF1 induction was significantly decreased (Figure 2A). Induction of another EBV immediate early gene, BRLF1, was also significantly inhibited by clozapine (Figure 2B). To determine if the reduction in these viral immediate early genes by clozapine affects the expression of a downstream lytic gene, levels of the BMLF1 gene that encodes an mRNA export factor were measured [10,11]. Expression of BMLF1 was induced in cells treated with butyrate, as expected. The addition of clozapine plus butyrate decreased BMLF1 expression to levels comparable to untreated cells (Figure 2C).

### 3.2. Dose-Dependent Inhibition by Clozapine

**Figure 3:**
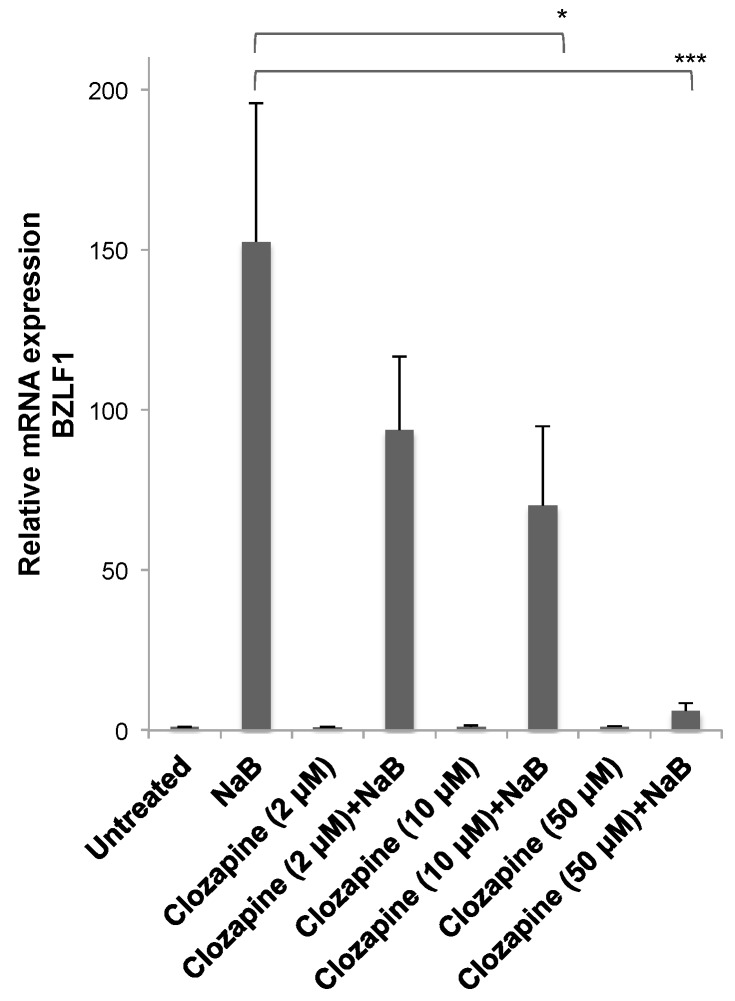
Inhibition of Epstein–Barr virus (EBV) lytic reactivation by clozapine was dose dependent. Clozapine (2, 10, and 50 μM) was tested in the presence and absence of NaB (3 mM) for the effects on BZLF1 expression in HH514-16 cells. Values are the average of seven biological replicates. There was no significant difference between untreated cells and cells treated with only clozapine at any concentration. Differences in BZLF1 expression comparing cells treated with butyrate in the absence and presence of clozapine are marked with * for *p*-value < 0.05 and *** for a *p*-value < 0.001.

The effects of varying concentrations of clozapine on the reactivation of EBV into the lytic cycle were tested. Clozapine at 2, 10, or 50 μM did not induce BZLF1 viral gene expression in the HH514-16 Burkitt lymphoma cells compared to untreated cells (Figure 3). When added with butyrate, lower concentrations of clozapine (i.e., 2 or 10 μM) inhibited BZLF1 expression by ~40%–50% compared to the level reached in cells treated with only NaB. Clozapine at 50 μM inhibited lytic reactivation by NaB by >95% compared to the reactivation seen with NaB alone.

**Figure 4:**
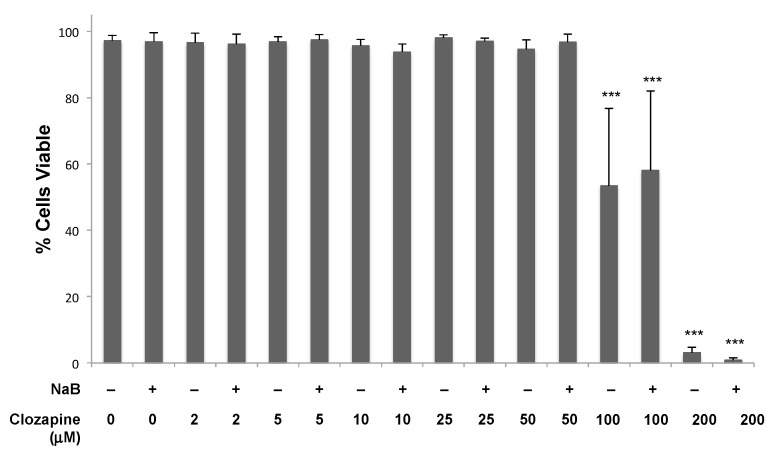
Cells remained viable when treated with 50 μM clozapine for 24 h. Clozapine was tested at concentrations from 2–200 μM in the presence and absence of NaB (3 mM) for the effects on the viability of Burkitt lymphoma cells. Data from four or more biological replicates were averaged, and error bars represent the standard deviation. Conditions are not marked unless significantly different than untreated cells. Differences with a *p*-value < 0.001 are denoted with ***.

The concentrations of clozapine that inhibited EBV lytic reactivation, up to 50 μM, did not limit cell growth or the percentage of dead cells when treated for 24 h (Figure 4). With 50 μM clozapine, cells remained >93% ± 4% viable (*n* = 6) after 48 h and 88% ± 3% viable (*n* = 3) after 72 h of treatment. Toxicity was observed within 24 h when the clozapine concentration reached 100 μM. Cell toxicity with 100 μM clozapine varied widely among experiments, but the average of 12 replicates resulted in ~40% cell death. When the clozapine concentration reached 200 μM, nearly all of the cells were dead after 24 h in all experiments.

### 3.3. Clozapine Decreased EBV Lytic Induction by dAzaC and TPA

**Figure 5:**
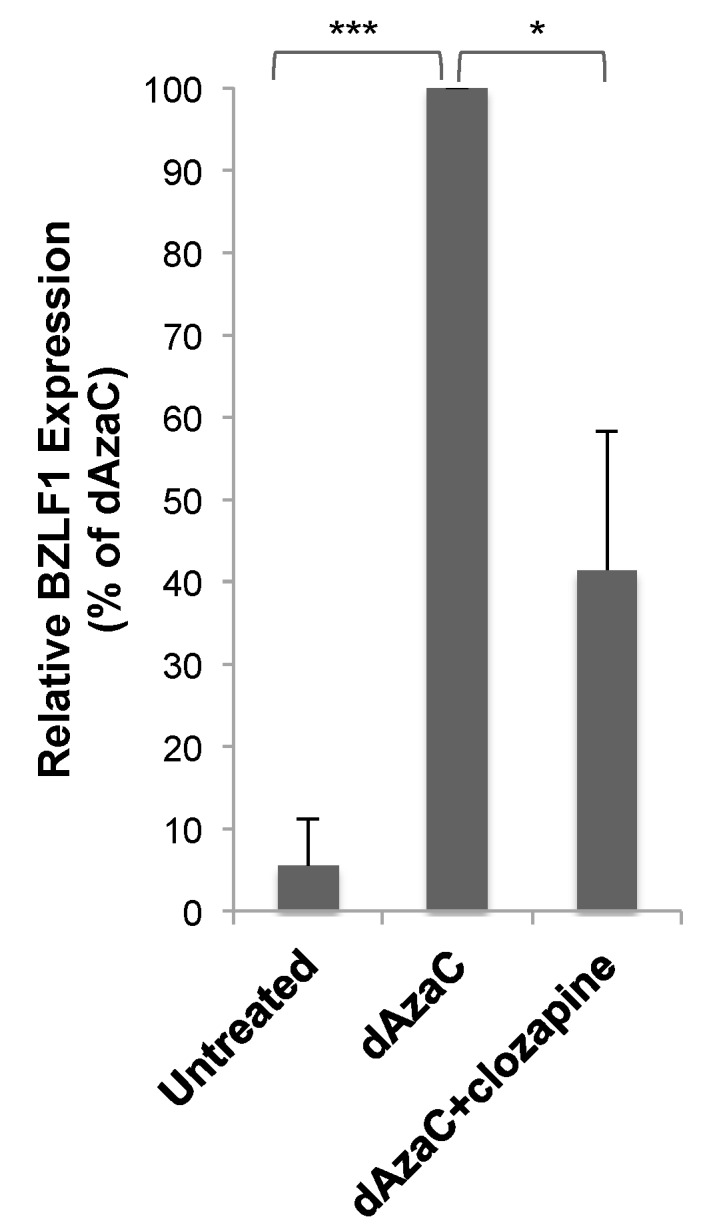
Clozapine decreased EBV lytic BZLF1 expression induced by 5-aza-2’-deoxycytidine (dAzaC). BZLF1 expression was measured in HH514-16 cells after treatment for 24 h with dAzaC (10 μM) alone or combined with clozapine (50 μM) and compared to untreated cells. The average of six biological replicates was plotted as a percentage of BZLF1 expression induced by dAzaC. *p*-value < 0.05 is denoted with *, *p*-value < 0.001 with ***.

Like butyrate, 5-aza-2’-deoxycytidine (dAzaC) also induces lytic gene expression in HH514-16 cells [3]. Sold under the drug name Decitabine, dAzaC is a DNA methyltransferase inhibitor that is thought to activate EBV by a different mechanism than butyrate [1]. dAzaC (10 μM) was not as potent an activator of BZLF1 expression (~40-fold) as butyrate in HH514-16 cells, but activated the expression of BZLF1 significantly compared to untreated cells (Figure 5). The addition of clozapine (50 μM) at the same time as dAzaC resulted in a 60% decrease in BZLF1 expression compared to dAzaC alone. Clozapine decreased EBV lytic reactivation stimulated by two different lytic inducing agents, but the effectiveness varied. This may have been due to the different mechanisms used by the inducing agents and the shorter length of exposure time required for dAzaC to induce the EBV lytic cycle [12].

**Figure 6:**
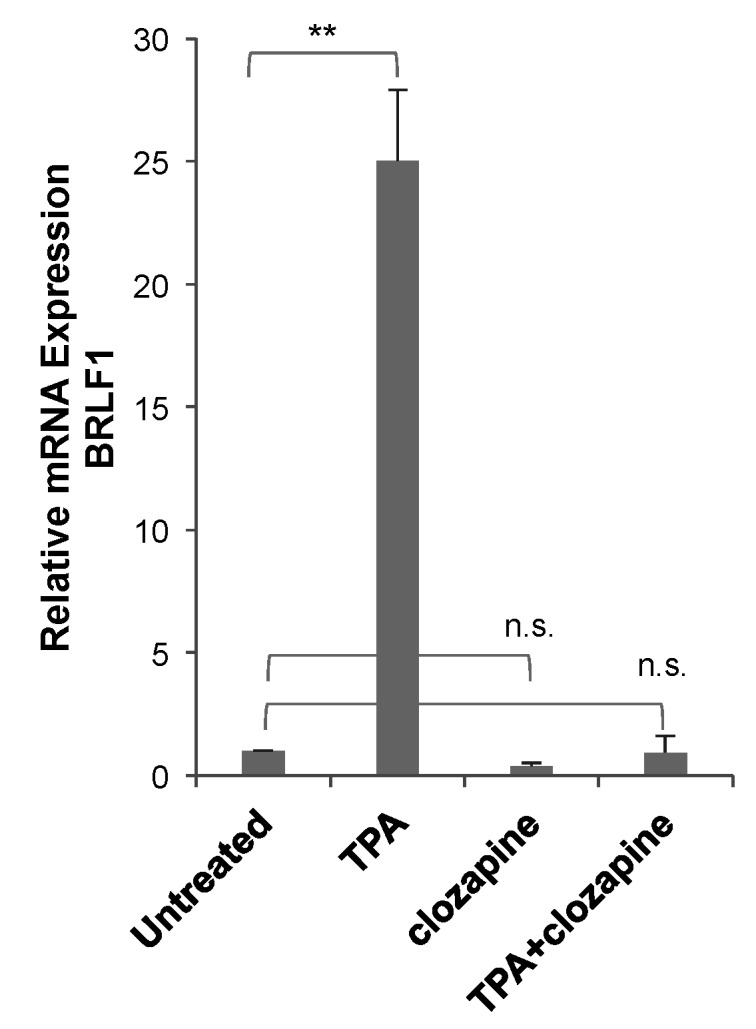
Clozapine inhibited EBV lytic BRLF1 expression in Raji cells. BRLF1 expression was measured in Raji cells after treatment with TPA (20 ng/mL) for 24 h in the absence and presence of clozapine (50 μM). The average BRLF1 expression of three or more biological replicates for each of the treated conditions was compared to untreated cells. Treated conditions are marked not significantly different (n.s.) or different with *p*-value < 0.01 (**) compared to untreated.

To determine the effectiveness of clozapine as an inhibitor in a separate EBV^+^ cell line, lytic reactivation was tested in Raji cells—a Burkitt lymphoma cell line with a different genetic background than HH514-16 cells. The lytic cycle was induced in the Raji cells by the addition of TPA (20 ng/mL) and detected by the expression of the EBV BRLF1 mRNA (Figure 6). When the cells were treated with clozapine (50 μM) and TPA for 24 h, the induction of BRLF1 expression was blocked. Raji cells treated with clozapine alone showed a 60% decrease, though not statistically significant, in basal levels of BRLF1 expression compared to untreated cells. These results provide evidence that clozapine inhibits EBV lytic reactivation by different classes of inducing agents and in different cell lines.

### 3.4. Metabolites of Clozapine

**Figure 7:**
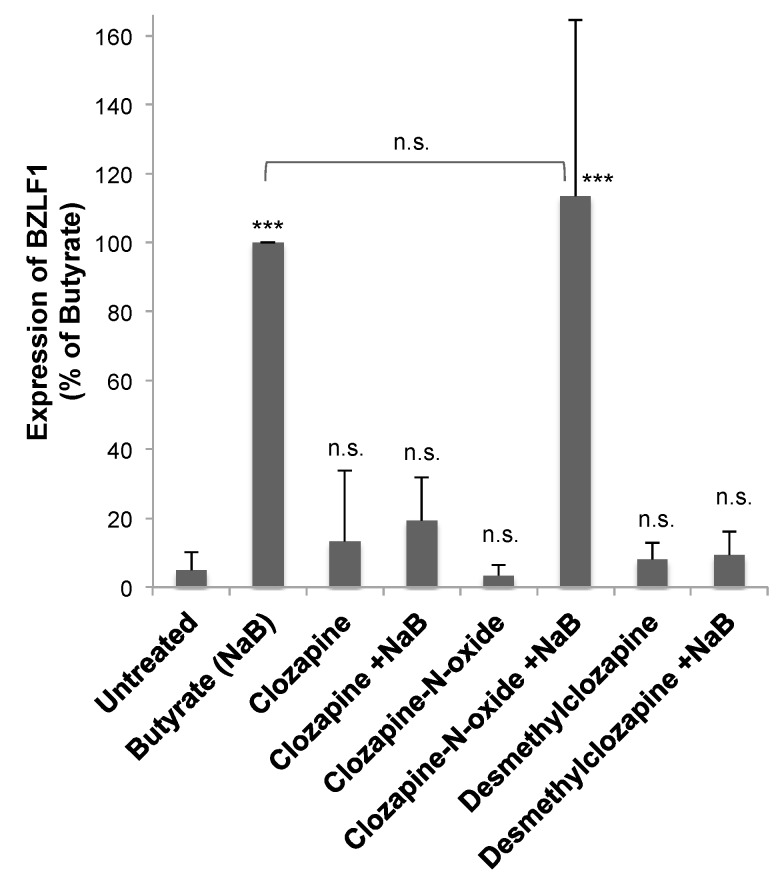
Desmethylclozapine, a metabolite of clozapine, inhibited EBV lytic reactivation, but clozapine-N-oxide did not. HH514-16 cells were treated with clozapine (50 μM), clozapine N-oxide (50 μM), or desmethylclozapine (50 μM) for 24 h in the presence and absence of butyrate (NaB; 3 mM). EBV lytic reactivation was measured by the expression of BZLF1. The averaged data are plotted as a percent of the BZLF1 expression induced by NaB. Data represent the average and standard deviation of five biological replicates. Changes from untreated are marked n.s for not significant, *p*-value < 0.001 is denoted with ***, and *p*-value < 0.05 with *. The NaB and NaB+clozapine-N-oxide were not significantly different.

Two of the major metabolites of clozapine—clozapine-N-oxide (CNO) and N-desmethylclozapine (NDMC; norclozapine) [13]—were tested to determine if the effects of clozapine on EBV lytic reactivation would be altered as the drug was metabolized. Neither clozapine metabolite by itself had any effect on basal levels of BZLF1 expression in HH514-16 Burkitt lymphoma cells (Figure 7). Cells were then treated with butyrate and CNO at 50 μM—the same concentration at which clozapine inhibited BZLF1 expression (Figure 2 and Figure 3). The induction of BZLF1 expression by NaB was the same in the absence or presence of CNO, demonstrating no inhibitory effect by CNO (Figure 7). However, when combined with butyrate, NDMC (50 μM) decreased BZLF1 expression by 90% compared to butyrate alone. Therefore, CNO did not decrease BZLF1 expression, but clozapine and its metabolite NDMC did inhibit EBV lytic gene expression.

## 4. Discussion

### 4.1. Concentrations of Clozapine in Therapeutic Use

Clozapine, shown here to inhibit expression of EBV lytic genes, is an antipsychotic drug used to treat schizophrenia. The standard dosing for patients is 300–600 mg of clozapine per day [14]. The recommended therapeutic range for clozapine plasma levels range from 350–550 ng/mL for effective treatment. The actual concentrations vary by patient, with factors such as weight and whether the patient smokes influencing this greatly. Studies of patients taking 400 mg/day of clozapine have measured blood concentrations of 40–1911 ng/mL and 84–1088 ng/mL [15]. The maximum plasma level recommended varies between 600 and 2000 ng/mL [16]. The clozapine concentration that inhibited EBV was 50 μM (Figure 3), which is ~8–16-fold higher than plasma concentrations in patients. In experiments conducted in vitro, concentrations of clozapine up to 50 μM had no effect on the viability of HH514–16 Burkitt lymphoma cell line (Figure 5). In another study that used a modified tetrazolium assay to assess the viability of U-937 cells from a patient with histiocytic lymphoma, clozapine had no effect on cell survival after 24 h of treatment with 6250 ng/mL (19 μM) clozapine, and 80% of cells survived when exposed to 12,500 ng/mL (~40 μM) clozapine [17]. No toxicity was observed by clozapine at >50 μM in neutrophils, monocytes, or HL-60 human leukemia cells [18].

### 4.2. Metabolites of Clozapine

The metabolism of clozapine is catalyzed by the cytochrome P450 enzymes in the liver into two main metabolites: clozapine-n-oxide (CNO) and N-desmethylclozapine (NDMC). NDMC is found in patient plasma at concentrations similar to clozapine, while CNO is much less [13]. CNO is pharmacologically inactive, but has the potential to reverse-metabolize into its parent compound clozapine [19]. No therapeutic benefits of NDMC have been demonstrated for the treatment of schizophrenia [20], but it does have biological effects. While clozapine is an antagonist of the dopamine D_2_ receptor, NDMC is a partial agonist in some assays [21]. NDMC is an allosteric agonist at the muscarinic M1 receptor. The muscarinic agonist activity of NDMC can potentiate N-methyl-D-aspartate (NMDA) receptor currents [22]. NDMC has a higher affinity for 5-HT_1C_ and 5-HT_2_ receptors than clozapine, while CNO is less potent [23]. NDMC is more effective than clozapine as a partial agonist of the 5-HT_1C_ receptor [21]. At higher concentrations, clozapine and NDMC, but not CNO, also antagonize the GABA_A_ receptor [24]. Overall, clozapine and NDMC are more biologically active than CNO, which correlates with the observed effects on EBV lytic reactivation where clozapine and NDMC inhibited EBV, but CNO did not (Figure 7).

### 4.3. The Effects of Clozapine on Immune Cells

One of the most potentially critical side effects of clozapine is agranulocytosis—a reduction in granule-containing white blood cells, particularly neutrophils. Due to the potential for agranulocytosis and a high risk of infection, patients taking clozapine require long-term hematology monitoring. A number of mechanisms for the clozapine-induced agranulocytosis have been explored [25,26]. In macrophages, clozapine affects adhesion, phagocytosis, and reactive oxygen species production [27]. Clozapine also alters cytokine production in macrophages [27]. Effects of clozapine on cytokine production have been observed in a number of studies on peripheral blood mononuclear cells or whole blood and in patients with schizophrenia, though reported results have varied, and even contradicted, possibly due to varying cell sources and treatment conditions [28]. Clozapine suppresses interferon-γ production in peripheral blood mononuclear cells and inhibits Th1 cell differentiation [29]. Clozapine inhibits the production of the T-bet transcription factor and enhances mRNA expression of STAT6 and GATA3 [29]. Whole-genome analysis using the T lymphocyte cell line JM-Jurkat treated with clozapine revealed changes in expression of hundreds of mRNAs and miRNAs involved in a number of cellular processes, including cellular metabolism and oxidative stress [30]. The effects of clozapine on B cells have been less well studied.

### 4.4. Mechanism of Action

The first-generation, or typical, antipsychotic drugs are high-affinity antagonists of the dopamine D_2_ receptor. However, the extrapyramidal symptoms (EPSs), such as involuntary movement and muscle control, caused by typical antipsychotics have been attributed to potent dopamine antagonism [31]. The second-generation, atypical, anti-psychotic drugs such as clozapine have less affinity for the D_2_ receptors and result in reduced EPS side effects. Atypical drugs also target other dopamine receptors (D_1_, D_3_, D_4_), the 5-HT_1A_, 5-HT_2A_, 5-HT_2C_, 5-HT_6_, and 5-HT_7_ receptors for serotonin (5-hydroxytryptamine), as well as muscarinic, adrenergic, and histamine receptors [32]. Clozapine has a high affinity for the serotonin 5-HT_2A_ receptor [33,34]. A theory on the effectiveness of atypical antipsychotics relates a higher ratio of a drug’s affinity for the 5-HT_2A_ receptor compared to the dopamine D_2_ receptor. The 5-HT_1A_ receptor may also play a role in the antipsychotic effect, as the receptor is stimulated by clozapine and other antipsychotic drugs [35]. Serotonin is not only active in the nervous system, but also affects the immune system. Serotonin increases the mitogen-stimulated proliferation of B-cells, which is dependent on the serotonin 5-HT_1A_ receptor [36]. The 5-HT_3A_ receptor is expressed on B-cells and is differentially expressed in diffuse large B-cell lymphomas (DLBCL) compared to non-neoplastic B cells [37]. The serotonin transporter (SERT) is also expressed on B cells. Culturing Burkitt lymphoma cell lines with serotonin leads to increased serotonin uptake by SERT, decreased DNA synthesis, and apoptosis of Burkitt lymphoma cells, including in EBV-positive cells [38].

The mechanism of action of VPA as an inhibitor of EBV lytic reactivation in lymphoma cells is not known [4,39]. VPA is known to have a number of effects in neurons, however, which of these roles is responsible for the clinical effects of this drug in neurological disorders is uncertain [40]. The proposed mechanisms of VPA and clozapine are not similar, and they do not have a similar chemical structure, so it is possible that the two drugs affect EBV lytic reactivation in different ways. The cellular targets of antipsychotic, anti-epileptic, and mood-stabilizing drugs will continue to be explored in cells infected by EBV.

### 4.5. Effect of Clozapine and Its Metabolites on Other Viruses

The clozapine metabolite N-desmethylclozapine was found to inhibit replication of dengue virus (DENV) [41]. The inhibition of N-desmethylclozapine was specific to DENV. It did not have an effect on other flaviviruses (i.e., Japanese encephalitis virus, West Nile virus), or on the RNA viruses respiratory syncytial virus and rotavirus. Inhibition occurred at an early step in the viral life cycle prior to viral replication. Two other metabolites of clozapine, 8-OH-deschloro-clozapine and 8-OH-desmethylclozapine, inhibit human immunodeficiency virus (HIV) type 1 [42]. Neither clozapine, the primary compound, nor desmethylclozapine show any antiviral effects, suggesting that inhibition of HIV is due to the metabolism of clozapine. Clozapine (30 μM) decreased the infection of a human glial cell line by the human polyomavirus JC virus by approximately one-half [43]. The human endogenous retroviruses (HERVs) are associated with schizophrenia and other neurological diseases. Clozapine had no significant effect on the transcription of HERVs in three types of brain cell lines, though VPA upregulated the transcription of many HERVs [44]. Valpromide, an anti-epileptic drug that inhibits EBV lytic reactivation [5], had no effect on vesicular stomatitis virus infection [45]. Valpromide and a structurally-related molecule valnoctamide inhibited infection and replication of the human herpesvirus cytomegalovirus in cell culture, and increased the survival rate of mice infected with mouse cytomegalovirus [45].

In conclusion, the repurposing of antipsychotic drugs as antivirals has the potential for therapeutic use. Understanding how clozapine inhibits EBV may lead to the discovery of cellular pathways that regulate the latent–lytic switch of the virus. Future studies will be aimed at determining the effects of other antipsychotic drugs on EBV lytic reactivation and identifying common molecular targets in cells that may provide information about the mechanisms important in neurological disease and for the viral life cycle.
